# Diagnostic value of RT-PCR for detection of tick-borne encephalitis virus RNA in cerebrospinal fluid

**DOI:** 10.1007/s10096-025-05389-x

**Published:** 2026-01-12

**Authors:** Hedvig Glans, Sofia Bartholdsson, Nina Lagerqvist, Hampus Nord, Ola Blennow, Jakob Morén, Gabriel Westman, John Karlsson Valik, Jonas Klingström, Sara Gredmark-Russ

**Affiliations:** 1 Department of Infectious Diseases, Karolinska University Hospital, Stockholm, Sweden; 2 Department of Medicine Huddinge, Center for Infectious Medicine, Karolinska Institutet, Alfred Nobels Allé 8, Huddinge, 141 52 Stockholm, Sweden; 3 Department of Microbiology, Public Health Agency of Sweden, Solna, Sweden; 4 Department of Infectious Diseases, Södersjukhuset, Stockholm, Sweden; 5 Department of Infectious Diseases, Capio St Göran´S Hospital, Stockholm, Sweden; 6 Infection Medicine, Department of Medical Sciences, Uppsala University, Uppsala, Sweden; 7 Department of Infectious Diseases, Uppsala University Hospital, Uppsala, Sweden; 8 Department of Medicine Solna, Karolinska Institutet, Stockholm, Sweden; 9 Division of Molecular Medicine and Virology, Department of Biomedical and Clinical Sciences, Linköping University, Linköping, Sweden; 10 The Laboratory for Molecular Infection Medicine Sweden (MIMS), Umeå, Sweden

**Keywords:** TBE, TBEV, PCR, Immunosuppressed patients/immunosuppressive treatment

## Abstract

**Purpose:**

Tick-borne encephalitis (TBE) diagnosis is mainly based on serology, which however can be difficult to interpret in previously TBE-vaccinate patients and in immunosuppressed patients. The objectives of this study were to investigate if the detection of tick-borne encephalitis virus (TBEV) RNA in cerebrospinal fluid (CSF) can be used to diagnose TBE, and to assess the relation of TBEV RNA positivity to clinical characteristics and disease severity.

**Methods:**

A retrospective study was conducted in TBE patients from Region Stockholm and Uppsala, Sweden, 2015–2020. CSF-samples were analysed for TBEV RNA using RT-PCR. Data on clinical parameters, retrieved from medical records, were analysed based on TBEV RNA positivity in CSF.

**Results:**

CSF from 36 TBE patients were analysed for the presence of TBEV RNA. TBEV RNA was detected in twenty of these patients (56%). Patients with detectable TBEV RNA in CSF had a more severe disease, with a higher rate of intensive care and assisted ventilation treatment. Overall, the mortality was 40% in patients with detectable TBEV RNA in CSF. In a subgroup of 8 patients with immunosuppressive treatment and detectable TBEV RNA in CSF, 7 (88%) had a fatal outcome.

**Conclusion:**

TBEV RT-PCR can be used as a complement for diagnostics in patients with underlying comorbidities involving the immune system or immunosuppressive treatment. The presence of TBEV RNA in CSF may correlate to severe infection and an increased risk of fatal outcome.

## Introduction

The flavivirus tick-borne encephalitis virus (TBEV) causes tick-borne encephalitis (TBE), a disease affecting the central nervous system (CNS). TBEV is mainly transmitted to humans by the bite of an infected hard tick, mainly *Ixodes ricinus* and *I. persulcatus*, and can also be transmitted via unpasteurized dairy products [1]. There are five different subtypes of TBEV; European (TBEV-Eu), Far Eastern (TBEV-FE) and Siberian (TBEV-Sib), and the two recently discovered subtypes Baikalian TBEV [2] and Himalayan TBEV [3]. TBEV-Eu is present in Europe, including Sweden [4]. In Finland and the Baltic region, TBEV-Sib have also been detected [5, 6]. The incidence of TBE is increasing in Europe [7, 8]. During 2023, Sweden reported a record-high number of 595 cases [9]. There is to date no available treatment for TBE, but available vaccines have an estimated field effectiveness of 95–99% [10]. The World Health Organization recommends offering TBE vaccination in areas where the average pre-vaccination incidence of clinical disease is ≥ 5 cases/100 000 population per year. Immunization should also be targeted to individuals performing certain outdoors activities in lower incidence areas, and to travellers that will engage in extensive outdoor activities in endemic areas [11].

TBEV infection can present with a wide range of symptoms, from asymptomatic or mild infection to severe CNS infection with neurological manifestations, typically meningitis, meningoencephalitis and meningoencephalomyelitis [12]. Most of the patients present with a biphasic course of disease, with a first phase of milder flu-like symptoms followed by a symptom free interval before the second phase with neurological symptoms [13–15]. This biphasic course is sometimes absent, especially in previously vaccinated individuals and in the elderly [15]. The case-fatality rate of TBEV-Eu is rather low, 0.75–2% [13, 16], whereas the morbidity is significant with long-term sequelae observed in approximately one third of the cases [13, 15, 17–19]. The more severe the TBE is, the higher the risk of sequelae and an incomplete recovery after the acute disease [17, 20, 21]. The majority of the TBE cases are diagnosed based on serology, through detection of TBEV-specific IgM and IgG in sera [22, 23]. Serological assays in previously TBE-vaccinated patients often need more interpretation and complementary samples to correctly diagnose the infection [24]. Detection of TBEV with real time (RT)-PCR is possible, but the virus is often only detected during the first phase of the infection, in which patients often suffer from milder disease, with unspecific symptoms, usually not requiring medical attention [25–27]. However, the virus may also be present during the second, meningoencephalitic phase of the disease. For example, Steininger et al., analysed TBEV RNA in samples from 89 cases of TBE: while none of the immunocompetent patients had detectable TBEV RNA in serum or CSF, two immunocompromised patients, treated with the B cell-depleting antibody rituximab (anti-CD20), were positive for TBEV RNA in CSF [28]. The detection of TBEV, by RT-PCR, in blood and urine in immunocompromised patients suggest possibilities to improve diagnostics in this patient group [29].

The aim of the present study was to investigate when and in which patient groups analysis of TBEV RNA in CSF can be beneficial, by relating underlying diseases, the TBEV-disease severity and clinical outcome to the presence of TBEV RNA.

## Material and methods

### Study population and design

Between 2015 and 2020, patients aged ≥ 18 years in Region Stockholm and Region Uppsala, Sweden, with confirmed TBE in whom CSF had been analysed for TBEV RNA at the Public Health Agency of Sweden, were included in the study. TBE was defined according to the ECDC [22, 23] with at least one of the following laboratory criteria for case confirmation: TBE specific IgM and IgG antibodies in blood, TBE specific IgM antibodies in CSF, seroconversion or four-fold increase of TBE specific antibodies in paired serum samples, detection of TBE viral nucleic acid in a clinical specimen or isolation of TBEV from clinical specimen.

In the serum samples obtained at admission, 20 out of the 36 patients (56%) were positive for TBE specific IgM and IgG. Out of the patients that were not positive for TBE specific IgM at admission 10 patients seroconverted, defined as the appearance of IgM and/or IgG or a titer increase in a follow up sample. All patients with no detection of TBEV RNA in the cerebrospinal fluid were either IgM and IgG positive at submission or seroconverted. In the patient group that were TBEV RNA positive in cerebrospinal fluid, one patient had no serological response, and 6 of the patients were IgG positive, with no IgM, and no follow up serological samples were obtained.

Vaccinated patients were categorised into completely vaccinated (vaccinated according to the recommended schedule in Sweden during a specific year), or incompletely vaccinated (vaccination that did not adhere to the recommended vaccine schedule), thoroughly presented in [30]. The standard vaccination schedule was initially 2 primary doses (0, 1–2 months), followed by additional doses at 5–12 months and 3 years, and further doses thereafter every 5 years, an extra priming dose at month 3 was recommended for all individuals aged 60 years or older. From 2016, an extra priming dose at month 3 was recommended for all individuals with immunosuppressive therapy and from 2018 for all individuals aged 50 years or older.

Clinical data were retrieved retrospectively from medical records and covered basic clinical and laboratory data as well as the clinical outcome of the patients according to a standardized protocol. The Glasgow Coma Scale (GCS) were used to evaluate to assess the level of consciousness, to describe the extent of impaired consciousness in the patient [31].

Ethical approval was obtained from the Regional and Central Ethical Review Board in Sweden, Dnr 2018/2107–31/2, 2019–03493 and 2024–08310-02.

### Laboratory methods

TBEV RT-PCR was performed as part of the diagnostics at the Public Health Agency of Sweden. Viral RNA was extracted from 200-µL clinical specimens of cerebrospinal fluid using the MagDEA Dx SV reagent kit and the magLEAD automated extraction instrument (Precision System Science) according to the manufacturer’s protocol. Two RT-PCR assays were used: one modified from a published assay [32] targeting the conserved 5’ region of the TBEV genome (Assay-1) and one targeting the NS3 gene (Assay-2). A sample was considered positive for TBEV RNA if testing positive in at least one assay. Primers and probes (5’−3’) for Assay-1: forward GCGGTTCTTGTTCTCCCTGA, reverse CACACATCACCTCCTTGTCAGAC, and probe FAM-CCACCATCACCCAGAC-MGB-NFQ and Assay-2: forward TGGACATGCACCCAGGCT, reverse GCTCCATTTCYTTGAGYACCAC, and probe FAM-CACAGAGTCCTCCC-MGB-NFQ, were from Applied Biosystems.

The TBEV RT-PCR assays were carried out in 25-µL reaction mixtures containing 0.9 µM of each primer and 0.2 µM probe, 5 µL of RNA template, TaqMan Fast Virus 1-Step Master Mix (Applied Biosystems), and RNAse/DNAse-free water (Life Technologies, Thermo Fisher Scientific) in a StepOne Plus real-time PCR system (Applied Biosystems) with cycling parameters as follows: 50 °C for 5 min, 95 °C for 20 s followed by 45 cycles at 95 °C for 3 s and at 60 °C for 30 s.

### Statistical analysis

Statistical analysis was conducted using R. Differences in clinical characteristics were analyzed in relation to the presence of TBEV RNA in CSF. Categorical variables were expressed as proportions with differences analysed using Fisher’s exact test. Continuous variables were presented as median (IQR), and differences in distribution between groups were analysed using the Wilcoxon rank sum test. A p-value below 0.05 was considered statistically significant.

## Results

Thirty-six patients aged ≥ 18 years in Region Stockholm and Region Uppsala with confirmed TBE and CSF samples analysed for TBEV RNA between 2015 and 2020 were included in the study. Fourteen (39%) of the patients in the study were women. In total, 20 (56%) of these patients had detectable TBEV RNA in the CSF, 10 of them were women. The median age was similar in the TBEV RNA negative and positive group (Table 1). Two-thirds of the included patients were previously vaccinated, completely or incompletely, with similarly distribution between the TBEV RNA negative and positive (Table 1). In three of the patients the vaccination status was unknown.

**Table 1:** Demographic and basic clinical data of 36 patients, in Stockholm and Uppsala, Sweden, with tick-borne encephalitis and TBEV RNA analysed in cerebrospinal fluid, during 2015 to 2020. Divided by TBEV RNA result

	Total		TBEV RNA negative		TBEV RNA positive	
	*n*	%	*n*	%	*n*	%
	36	100	16	44	20	56
Sex						
Women	14	39	4	25	10	50
Age (years)						
Median (IQR)	59 (43–71)		59 (43–71)		59 (45–71)	
Previous TBE Vaccination						
Non-vaccinated	10	28	3	19	7	35
Incompletely vaccinated	12	33	6	38	6	30
Completely vaccinated	11	31	5	31	6	30
Underlying Comorbidity						
Any underlying comorbidity	23	64	9	56	14	70
Neurological disease	3	8	0	0	3	15
Haematological malignancy	4	11	1	6	3	15
Other malignancies	1	3	0	0	1	5
Inflammatory disease	9	25	3	19	6	30
Cardiovascular disease	10	28	5	31	5	25
Diabetes mellitus	2	6	1	6	1	5
Other	4	11	4	25	0	0
Immunosuppressive Therapy						
Any immunosuppressive therapy	11	31	3	19	8	40
Prednisolone equivalents*	7	19	2	13	5	25
Methotrexate	4	11	0	0	4	20
TNF-alpha inhibitors	3	8	2	13	1	5
Cytostatic	2	6	0	0	2	10
Rituximab	5	14	1	6	4	20
Other**	1	3	0	0	1	5

Any dose of prednisolone equivalents

^**^ Mesalazine and Azathioprine

Underlying comorbidities were common in both the TBEV RNA negative and positive patients, 56% and 70%, respectively (Table 1). Underlying diseases that were more common in the TBEV RNA positive groups included neurological diseases and diseases involving the immune system, such as haematological malignancy and inflammatory disease. In addition, 40% of the patients with detectable TBEV RNA in the CSF were on immunosuppressive treatment, as compared to 19% of the TBEV RNA negative patients (Table 1). Among patients on immunosuppressive therapy, four patients had been vaccinated against TBE, with two adhering to the recommended schedule and two that were vaccinated with only one dose of the vaccine (data not shown).

The interval between symptom onset and lumbar puncture was similar in both TBEV RNA positive and negative patients. This suggests that the timing of analysis for viral RNA did not account for the observed differences in the two groups (Table 2). TBEV RNA positive patients had a lower GCS (Median 8 versus 15, p-value < 0.001, Table 2) than TBEV RNA negative patients. Further, they had more admissions to the intensive care unit (ICU) (80% versus 19%, p-value 0.001), and more often required assisted ventilation (75% versus 19%, p-value 0.002) (Table 2). A total of 10 cases of myelitis were observed in our study population, of which eight patients were TBEV RNA positive (Table 2). Fifty percent of the patients was referred to rehabilitation after the infection, similar distribution in both TBEV RNA negative and positive group. We observed an in-hospital mortality of 31% (11 patients) whereof eight patients (73%) were positive for TBEV RNA in the CSF (p-value 0.277, Table 2).

**Table 2:** Clinical, laboratory, and radiological parameters, hospitalization and mortality of the acute phase of tick-borne encephalitis in 36 patients, analysed with TBEV RNA in cerebrospinal fluid. Divided by TBEV RNA result

	Total		TBEV RNA negative		TBEV RNA positive		*P*-value
	n	%	*n*	%	*n*	%	
	36	100	16	44	20	56	
Clinical Characteristic							
Biphasic course	11	31	6	38	5	25	0.483
Days to lumbar puncture from symptom onset (Median, IQR)	4.5 (2–8)		4.5 (2–8)		5 (2.3–8.5)		
Fever	36	100	16	100	20	100	NA
Headache	20	56	9	56	11	55	1.000
GCS (Median, IQR)	11 (5–15)		15 (13–15)		8 (4–11)		<.001*
Myelitis	10	28	2	13	8	40	0.134
Severity of disease							
Mild	3	8	3	19	0	0	
Moderate	13	36	9	56	4	20	0.002*
Severe	20	56	4	25	16	80	
CSF findings							
Pleocytosis**	36	100	16	100	20	100	NA
Mononuclear leukocyte count, cells/μL (Median, IQR)	86 (46–155)		76 (43–122)		108 (48–169)		0.301
Polymorphonuclear leukocyte count, cells/μL (Median, IQR)	14 (4–78)		13 (4–69)		23 (6–141)		0.425
Albumin, mg/L (Median, IQR)	428 (343–666)		372 (297–602)		564 (384–744)		0.086
Glucose, mmol/L (Median, IQR)	3.4 (3.1–4)		3.3 (3.2–3.9)		3.5 (3.2–4.2)		0.205
Lactate, mmol/L (Median, IQR)	2.5 (2.1–2.9)		2.2 (1.8–2.6)		2.7 (2.3–3.3)		0.023*
Radiology, other diagnostics							
MRI performed	32	89	13	81	19	95	0.303
Pathological MRI***	20	63	6	46	14	74	0.150
EEG performed	22	61	4	25	18	90	<.001*
Pathological EEG findings***	21	96	3	75	18	100	0.182
EEG findings associated with epileptic activity***	7	32	3	75	4	22	0.077
Inpatient care							
Hospitalised	35	97	15	94	20	100	0.444
Days in hospital (Median, IQR)	10 (3–17)		10 (5–15)		5 (3–19)		0.812
ICU admission	19	53	3	19	16	80	0.001*
Days in the ICU (Median, IQR)	16 (9–37)		31 (20–39)		16 (9–36)		0.655
Assisted ventilation	18	50	3	19	15	75	0.002*
Days with assisted ventilation (Median, IQR)	15 (8–33)		29 (19–38)		15 (8–30)		0.614
Rehabilitation							
Any rehabilitation****	18	50	8	50	10	50	1.000
Mortality							
Mortality	11	31	3	19	8	40	0.277

^**^ > 5 mononuclear or polymorphonuclear leukocyte count, cells/μL

^***^ Percentage of those who underwent the procedure

^****^ Inpatient care at a geriatric ward, outpatient visits to and inpatient care at a neurorehabilitation unit included

*MRI* Magnetic resonance imaging, *EEG* Electroencephalogram

Magnetic Resonance Imagining (MRI) was performed in most of the patients (89%), while electroencephalogram (EEG) was more often performed in TBEV RNA positive patients (90%) as compared to TBEV RNA negative patients (25%) (p-value < 0.001, Table 2). Pathological findings on MRI or pathological activity, including both epileptic activity and unspecific pathological findings on EEG, were similar between the groups (p-value 0.150 resp. p-value 0.182).

An exploratory subgroup analysis of the patients on immunosuppressive therapy (*n* = 11) and mortality revealed that eight patients had a fatal outcome, and that all but one of these patients had detectable TBEV RNA in the CSF. The immunosuppressive therapies in these patients included corticosteroids, methotrexate, TNF-inhibitors, cytostatic treatment, rituximab and other (lenalidomide, ibrutinib, mesalazine and azathioprine), as single usage or in combination. No statistical significance was observed between the different therapies and mortality (data not shown).

## Discussion

TBE is an emerging viral infection, with an increasing incidence in Europe [7, 8]. For patients with previous TBE vaccination and in certain groups of immunosuppressed patients the gold-standard method of serology may be difficult to interpret, highlighting the need of complementary methods for diagnostics. We report that TBEV RNA was detected in CSF in about half of the patients in this highly selected cohort, suggesting that RT-PCR can be of value in diagnostics of suspected TBE. Further, we report that TBEV RT-PCR-positive patients show a more severe TBE, as reflected by a lower GCS, more intensive care treatment, assisted ventilation and higher mortality in this group.

The diagnostics of TBE is currently mainly based on serology, but for the growing group of immunosuppressed patients and/or previously vaccinated patients, serological analysis has limitations. In these patient groups, repeated blood samplings or detection of TBEV neutralising antibodies in sera are necessary to confirm the diagnosis [33]. Detection of antibodies directed to TBEV non-structural protein 1 (NS1) can also be useful in previously vaccinated patients [34]. The detection of TBEV RNA in a clinical specimen is one of the criteria of the case definition from ECDC [22, 23], however, TBEV RNA detection is only rarely used in clinical settings. In one study, the detection of TBEV RNA in CSF was the only way to verify acute TBEV-infection in two immunocompromised patients [28]. Thus, to only rely on serological findings may pose an increased risk of missing a TBE diagnosis in immunosuppressed patients. The slow IgM antibody response in TBE vaccination breakthrough infections complicates the interpretation of TBE serology and follow-up sampling is often necessary for diagnosis [24, 33, 35]. TBEV RNA analysis may contribute to a faster TBE diagnosis.

One third of the patients in our cohort were on immunosuppressive treatment. These therapies may affect TBE vaccine efficacy, and they might impact viral clearance and disease severity. Among immunosuppressive treatments, both methotrexate and rituximab have been previously demonstrated to significantly affect the TBE vaccination efficacy and to cause an insufficient antibody response [36]. A delayed or less effective antibody response after TBE vaccination has also been observed in thymectomized patients and after solid organ transplantation [37–39]. In immunocompetent patients, TBEV is usually not detectable in sera and in CSF at the time of CNS disease and after the antibody response has developed [25]. The inability to produce an adequate antibody response in patients on immunosuppressive treatment may contribute to the high detection rate of TBEV RNA in CSF in this patient group. This is in line with a previous report where TBEV RNA was detected in the CSF in two seronegative patients treated with rituximab [28]. The high rate of detectable TBEV RNA in the group of immunosuppressed patients indicates that TBEV RT-PCR may be valuable for diagnostics in immunosuppressed patients with high clinical suspicion of TBE, especially in seronegative patients and in patients with inconclusive serology.

The patients with presence of TBEV RNA in CSF in our cohort had a more severe TBE, including lower GCS, more intensive care treatment, and assisted ventilation, compared to the patients with non-detectable TBEV RNA. Comparing our cohort to other TBE cohorts, demonstrates that our patients had more severe TBE, higher percentages of intensive care treatment, and higher mortality [13, 16, 40]. For example, in a cohort with TBE patients in Germany, 13.8%, received intensive care [14], and in a Swedish cohort 5.6% of the patients were treated in the ICU [40]. In general, the case fatality for TBE is rather low (< 2%) in immunocompetent individuals. Our recent data on a cohort of 703 TBE patients in Region Stockholm suggests significantly higher case fatality in immunosuppressed patients (~ 15%) [40]. In our present cohort, patients with immunosuppressive treatment and detectable TBEV RNA in CSF had an even higher mortality; 87.5%, which may suggest that viral replication ultimately may contribute to fatal outcome. In accordance with this, TBEV has been detected in brain tissue from human fatal cases of TBE [41].

Our study has several limitations. The study was designed to investigate if the detection of TBEV RNA in cerebrospinal fluid (CSF) can be used as a complement to serology to diagnose TBE, and further to study if the presence of TBEV RNA relates to clinical characteristics and disease severity in individuals with TBE in whom TBEV RNA already was analysed as a part of their clinical investigation. Given that RT-PCR for TBEV RNA is not a routine clinical assay, we had a selection bias resulting in inclusion of patients more difficult to diagnose and with a more severe disease as compared to general TBE cohorts. The studied cohort is thus a highly selected TBE cohort.

TBEV RNA analysis can be used for diagnostics in patients with underlying diseases involving the immune system or in patients on immunosuppressive treatment, and in patients where serology is not conclusive, for example in previously vaccinated patients. The presence of TBEV RNA in CSF may correlated to a more severe infection and an increased risk of fatal outcome in immunosuppressed patients. These findings can contribute to better diagnostic strategies and may contribute to further understanding of the pathogenesis of TBE.

## Data Availability

No datasets were generated or analysed during the current study.
